# Assessing amyloid PET positivity and cognitive function in Down syndrome to guide clinical trials targeting amyloid

**DOI:** 10.1002/alz.14068

**Published:** 2024-06-28

**Authors:** Sophia Krasny, Cynthia Yan, Sigan L. Hartley, Ben L. Handen, Julie K. Wisch, Anna H. Boehrwinkle, Beau M. Ances, Michael S. Rafii, the ABC‐DS consortium

**Affiliations:** ^1^ Scripps Research Institute La Jolla California USA; ^2^ Department of Neurology Washington University Saint Louis Missouri USA; ^3^ Waisman Center University of Wisconsin Madison Wisconsin USA; ^4^ Department of Psychiatry University of Pittsburgh Pittsburgh Pennsylvania USA; ^5^ Alzheimer's Therapeutic Research Institute Keck School of Medicine of University of Southern California San Diego California USA

**Keywords:** adults, Alzheimer's disease, amyloid, clinical trials, cognitive, dementia, Down syndrome, imaging, positron emission tomography

## Abstract

**INTRODUCTION:**

Trisomy 21, or Down syndrome (DS), predisposes individuals to early‐onset Alzheimer's disease (AD). While monoclonal antibodies (mAbs) targeting amyloid are approved for older AD patients, their efficacy in DS remains unexplored. This study examines amyloid positron emission tomography (PET) positivity (A+), memory function, and clinical status across ages in DS to guide mAb trial designs.

**METHODS:**

Cross‐sectional data from the Alzheimer Biomarker Consortium–Down Syndrome (ABC‐DS) was analyzed. PET amyloid beta in Centiloids classified amyloid status using various cutoffs. Episodic memory was assessed using the modified Cued Recall Test, and clinical status was determined through consensus processes.

**RESULTS:**

Four hundred nine DS adults (mean age = 44.83 years) were evaluated. A+ rates increased with age, with mean amyloid load rising significantly. Memory decline and cognitive impairment are also correlated with age.

**DISCUSSION:**

These findings emphasize the necessity of tailoring mAb trials for DS, considering age‐related AD characteristics.

**Highlights:**

- There is rapid increase in prevalence of amyloid beta (Aβ) positron emission tomography (PET) positivity in Down syndrome (DS) after the age of 40 years.
- Aβ PET positivity thresholds have significant impact on prevalence rates in DS.
- There is a significant lag between Aβ PET positivity and clinical symptom onset in DS.

## 1 BACKGROUND

Throughout life, there is a continuous production and clearance of amyloid beta (Aβ). However, the accumulation of Aβ has been associated with both early onset Alzheimer's disease (EOAD) and late onset Alzheimer's disease (LOAD). EOAD is linked to increased expression of the amyloid precursor protein (*APP*) gene, leading to elevated APP production, while LOAD is believed to result from imbalances in Aβ clearance and proteostatic mechanisms.^1^

In individuals with Down syndrome (DS), trisomy of chromosome 21 amplifies the expression of the *APP* gene, causing increased APP processing and Aβ overproduction, which subsequently accumulates.^2^, ^3^ Notably, nearly all individuals with DS develop Alzheimer's disease (AD) neuropathology by age 40, with a lifetime risk of dementia > 95%.^4^

Neurodegeneration in AD can be evaluated through biomarkers such as Aβ42/Aβ40 levels in cerebrospinal fluid (CSF) and plasma, positron emission tomography (PET) imaging of Aβ plaques, and PET imaging of tau protein neurofibrillary tangles. The amyloid cascade hypothesis, which links Aβ accumulation to the subsequent development of neurodegeneration and clinical symptoms in AD, is widely cited in the literature.^5^, ^6^, ^7^

Several studies have demonstrated significant similarities in AD biomarkers within the amyloid–tau–neurodegeneration (ATN) framework between individuals with DS and other forms of AD.^8^, ^9^, ^10^, ^11^ With recent US Food and Drug Administration approval of anti‐amyloid immunotherapies showing clinical efficacy, it is imperative to consider how measures of amyloid, memory performance, and clinical status can be integrated into efficient AD clinical trials in individuals with DS. Endpoints such as amyloid positivity on PET imaging, memory performance, and clinical status are routinely used to measure disease progression in such trials.^7^, ^12^

We aim to elucidate the relationships among amyloid PET positivity, amyloid load, memory performance, and clinical status in individuals with DS and how these factors influence outcomes in clinical trials targeting amyloid. Specifically, we investigate the prevalence of amyloid PET positivity at various Centiloid (Cl) cutoffs by age, mean amyloid load in Centiloids by age, mean scores on the modified Cued Recall Test (mCRT) by age, and clinical status by age.

Clinical trials of amyloid‐lowering monoclonal antibodies (mAbs) in the general population have shown a 27% to 36% reduction in the rate of cognitive decline, along with an 85% reduction in baseline amyloid levels as assessed by PET imaging.^13^, ^14^ These antibodies are approved for use in individuals with “early AD,” meeting clinical criteria for mild cognitive impairment (MCI) or mild dementia with confirmed elevated brain amyloid. MCI in the general population is characterized by memory impairment without functional decline, while mild dementia involves limited functional decline. Determining how amyloid prevalence and positivity thresholds can inform clinical trials of mAbs in the DS population^15^ is crucial.

AD progresses through preclinical, prodromal, and dementia stages, a sequence also observed in DS‐related AD in terms of clinical manifestation and biomarkers.^9^, ^12^ Memory alterations in individuals with suspected AD aid clinicians in identifying cognitive decline due to disease progression. In adults with DS, AD‐related cognitive decline can be distinguished from baseline intellectual disability using the mCRT. This validated measure of AD‐specific cognitive decline in adults with DS demonstrates excellent sensitivity and specificity in detecting prodromal AD.^16^, ^17^

The mCRT captures essential features of cognitive decline in the DS population and is positively associated with Aβ and tau PET imaging before clinical AD dementia onset in DS.^12^ Additionally, the mCRT total score distinguishes between adults with DS with MCI versus AD dementia.^16^

In this paper, we explore amyloid PET positivity prevalence across ages using varying thresholds, as well as memory performance, baseline intellectual disability, and clinical status, to better inform the design of clinical trials targeting amyloid in the DS population.

## 2 METHODS

### 2.1 Procedures

The Alzheimer Biomarker Consortium–Down Syndrome (ABC‐DS) enrolls adults with DS (≥ 25 years old) in a multisite study that collects longitudinal clinical, cognitive, imaging, and fluid biomarker data. For this analysis, only participants from the baseline data collection cycle (data released January 2020) who had a magnetic resonance imaging (MRI) and amyloid PET scan were included (DS, *n* = 409; Table 1). Informed consent or assent, when appropriate, was obtained from all participants and their legally authorized representatives when necessary. Study protocols were approved by the local institutional review boards of all ABC‐DS sites.

**TABLE 1:** Demographics of the participants in the ABC‐DS cohort.

Age in years (*n* = 409)	Mean	SD
	44.83	9.75
Female (n_tot_ = 409)	*N*	%_tot_
	205	49.165
Intellectual disability (n_tot_ = 409)	*N*	%_tot_
Mild	193	47.5
Moderate	146	35
Severe/profound	36	8
NA	42	10
mCRT total (*n* = 338)	M	SD
	27.35	10.60
Clinical status (n_tot_ = 376)	*N*	%_tot_
Cognitively stable	259	68
MCI	53	14
Dementia	45	11
Unable to determine	19	0.05
PET Aβ Cl (*n* = 225)	M	SD
	24.06 Cl	35.65 Cl

Abbreviations: Aβ, amyloid beta; ABC‐DS, Alzheimer Biomarkers Consortium–Down Syndrome; Cl, Centiloids; MCI, mild cognitive impairment; mCRT, modified Cued Recall Test; PET, positron emission tomography; SD, standard deviation.

Participants with DS are given a clinical AD status by a consensus committee with clinical training or extensive experience in evaluating dementia in individuals with DS. This committee is blind to imaging data but considers all available cognitive and informant‐reported data and considers medical history, lifetime intellectual disability level, and recent major life events to derive a consensus status of cognitively stable (CS), MCI, or dementia due to AD (DEM). If no consensus is reached, a status of “unable to determine” is given. The determination of CS, MCI, and DEM is based on criteria previously published by the ABC‐DS group.^18^ Briefly, classification of clinical status, that is, DEM, MCI, and CS, was determined during clinical consensus conferences at which information from available sources including medical, clinical, and cognitive testing was reviewed. Participants were classified into three groups. Participants were classified as CS if cognitive or functional decline was absent. An MCI‐DS diagnosis was given to individuals whose cognitive and functional capacity was below age‐corrected capacity. Neuropsychological assessment and informant report served as the basis for diagnosis criteria. Participants were categorized as having dementia (DEM) if clinicians found substantial progressive decline in individuals’ cognitive functioning and daily living skills. An unable‐to‐determine category was used to indicate observed decline in cognitive and functional ability, which could be attributed to life circumstances (e.g., staff changes) or conditions unrelated to AD (e.g., severe sensory loss, poorly resolved hip fracture, psychiatric diagnosis). Level of intellectual disability was determined using the Kaufman Brief Intelligence Test, 2nd edition (KBIT‐2).^18^

T1‐weighted MRI scans were collected for ABC‐DS participants on 3‐Tesla MR scanners and segmented into regions of interest using FreeSurfer 5.3‐HCP with identical quality control procedures. Participants underwent amyloid PET imaging using [11C]‐Pittsburgh compound B (PiB) or [18F]‐AV45 (florbetapir).^18^ All PET images were processed and aligned to the FreeSurfer MR segmentation using an established processing pipeline (PET Unified Pipeline; https://github.com/ysu001/PUP). PiB PET images were spatially normalized to the Montreal Neurological Institute 152 space (MNI152) via a DS‐specific PET template for PiB as previously described.^19^ Using gray matter cerebellum as a reference tissue, standardized uptake value ratio (SUVR) images were generated through voxel normalization of summed PET images. Regional SUVRs were calculated using the cerebellar cortex as the reference region and converted to Cl scale.^20^

**RESEARCH IN CONTEXT**

1. **Systematic review**: Positron emission tomography (PET) imaging studies in individuals with Down syndrome (DS) have revealed early and aggressive accumulation of amyloid beta (Aβ). Prevalence rates of Aβ PET positivity across age ranges in relation to memory performance and clinical status have not been reported.
2. **Interpretation**: Our findings present the first report of Aβ PET positivity in relation to memory performance and clinical status in adults with DS.
3. **Future directions**: Understanding Aβ PET positivity prevalence rates across ages using varying thresholds, as well as memory performance and clinical status, will help better inform the design of clinical trials that target amyloid in the DS population. Future work will look at other biomarkers of AD in DS.

Memory was assessed using the mCRT. The mCRT is composed of two phases: the learning phase and the testing phase. In the learning phase, participants were shown three cards, each with four pictures of objects, then were asked to name the objects. The testing phase was divided into two sections. In the first section, participants were asked to freely recall as many pictures as possible. This was followed by the cued recall phrase, where a category cue (e.g., “fruit” for picture of grapes) was given for objects missed during the free recall section. The free and cued recall scores were summed to create the mCRT total score. The testing phase immediately followed the learning phase and consisted of three trials, each requiring recall of all 12 items. Initially, participants were asked to recall as many items as possible. Approximately 1 minute was allowed for free recall, after which category cues were provided for missed items. One point was given for each item that was recalled correctly. The total number of items recalled across all three trials, combining free and cued recall, was selected as the primary measure of performance (range = 0–36). The mCRT total score demonstrates high sensitivity for detecting MCI and dementia^16^ and is associated with imaging biomarkers including PET Aβ before dementia onset^12^ in individuals with DS.

### 2.2 Statistical analysis

In this analysis, a dataset containing amyloid PET imaging results, mCRT scores, and clinical consensus classifications was processed and examined. The dataset was preprocessed by removing instances with missing values in each of the key variables. Next, the data were stratified into age groups, spanning 5 years each, starting with 25 to 30 years and going to 55 to 60 years of age.

The mean and standard deviation of the mCRT and Centiloid data for each age bin were computed using the NumPy library. A simple linear regression model was used to estimate changes to mean mCRT by age decade. We also included standard error of the mean (SEM) to correct for population deviance. Spearman rho was used to assess the correlation between age group and mCRT score.

The prevalence of MCI, DEM, and unable to determine within each age group was examined and compared. Spearman rho was used to assess the association between age group and clinical status. All statistical computations were conducted using Jupyter Notebook run on Python v3.9.13.

## 3 RESULTS

### 3.1 Prevalence of Aβ PET (A+) positivity by age and threshold

**FIGURE 1:**
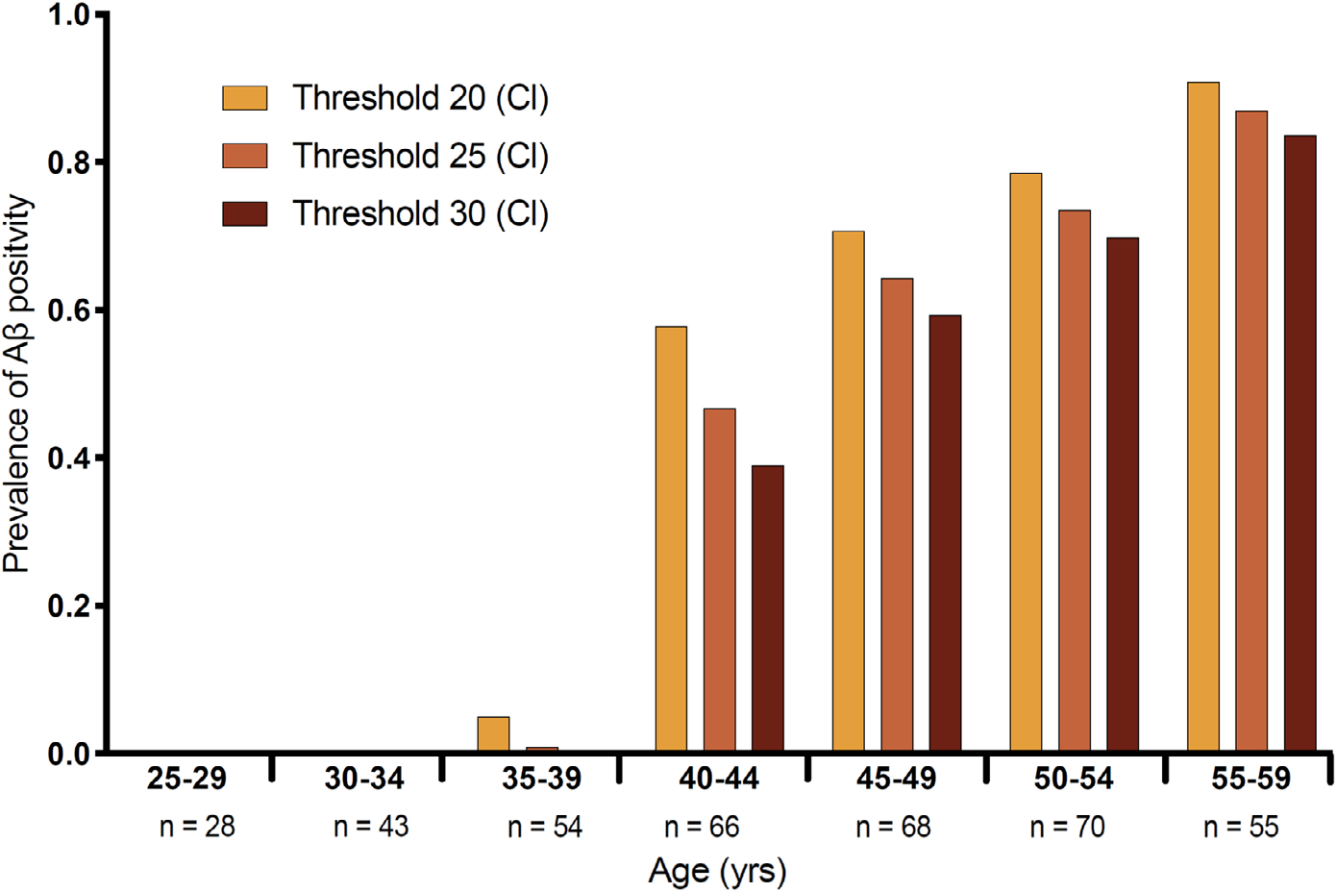
Relationship between age and proportion of sampled population positron emission tomography Aβ which meets threshold criteria at various threshold values (20, 25, 30 Cl). Aβ, amyloid beta; Cl, Centiloids

We estimated A+ prevalence across three thresholds (20, 25, and 30 Cl) by age group (Figure 1). These thresholds were selected based on results recently published in both sporadic AD and DS that indicate these thresholds may be predictive of progression.^21^, ^22^, ^23^, ^24^ The number of A+ participants increased with advanced age group, regardless of the threshold (ρ = 1.000, ρ = 0.9910, ρ = 0.9910 for 20, 25, and 30 Cl threshold, respectively). Notably, the largest increase in prevalence of A+ was observed between the age group of 35 to 39 years and 40 to 44 years. For participants aged 35 to 39 years, A+ prevalence was 5.01% (20 Cl), 0.85% (25 Cl), 0.11% (30 Cl). In contrast, for participants aged 40 to 44 years, A+ prevalence jumped to 57.81% (20 Cl), 46.72% (25 Cl), and 38.91% (30 Cl). The prevalence of A+ continued to increase with advanced age group such that among participants aged 55 to 59 years, positivity rates were 90.81% (20 Cl), 86.91% (25 Cl), and 83.64% (30 Cl).

### 3.2 Mean amyloid PET by age

**FIGURE 2:**
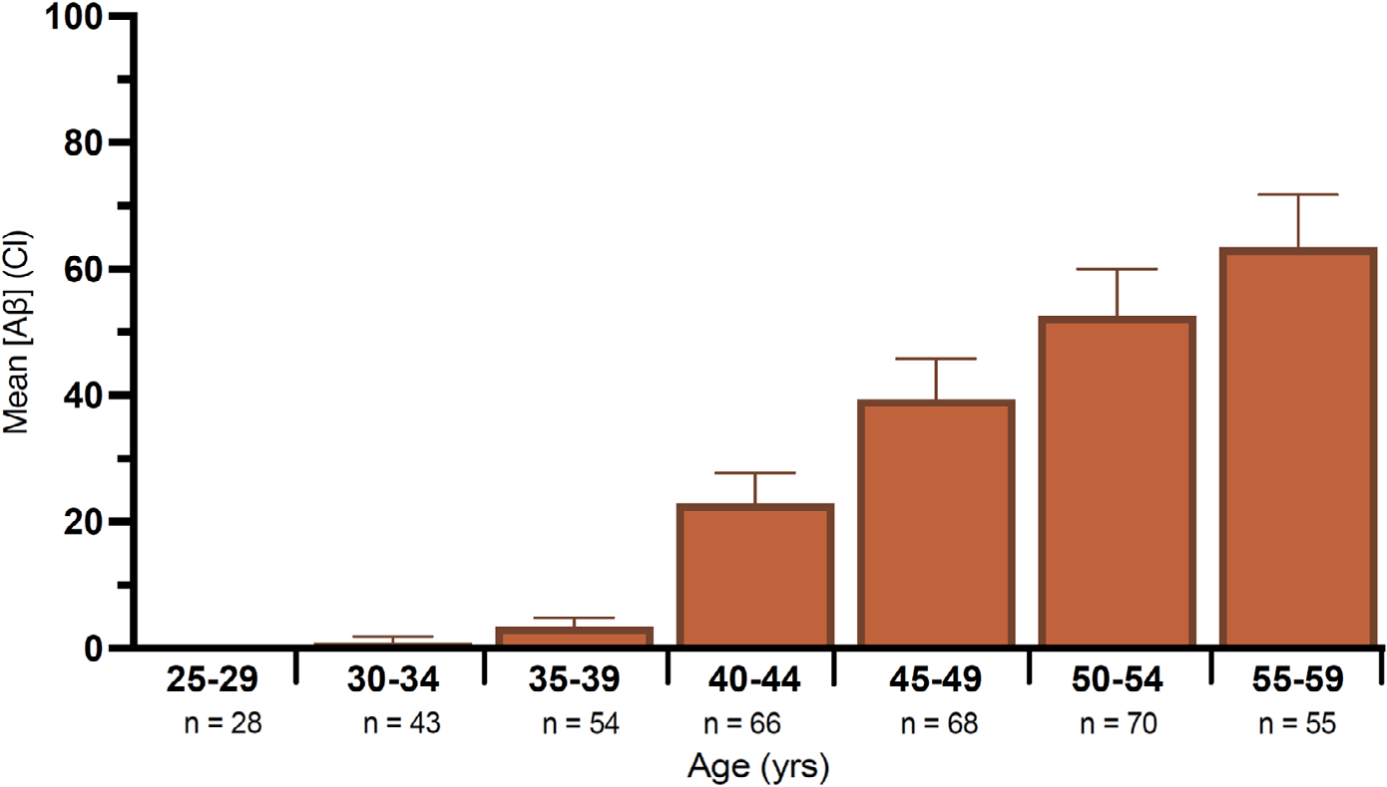
Mean positron emission tomography (Aβ) assessed in Cl by age. Error bars indicate standard error of the mean for each group. Aβ, amyloid beta; Cl, Centiloids

We estimated mean amyloid PET in Cls for each age group (Figure 2). For the 40‐ to 44‐year‐old group, mean amyloid PET was 22.92 Cl (standard deviation [SD] = 25.80, SEM = 0.88). This value nearly doubled for participants aged 45 to 49 years, who had a mean amyloid PET of 39.41 Cl (SD = 37.97, SEM = 1.08). Mean amyloid PET in Cl significantly increased across the age groups (ρ = 1.000, *P* = 0.0004).

### 3.3 Mean mCRT by age

**FIGURE 3:**
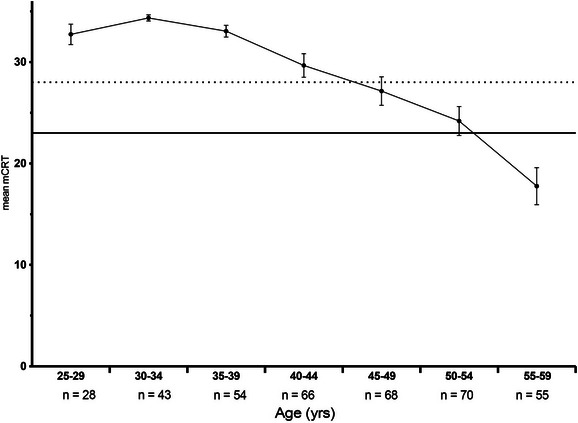
Mean modified Cued Recall Test (mCRT) scores by age. Error bars indicate standard error of the mean. Clinical diagnostic criteria are met for mild cognitive impairment when mCRT score = 28, while mCRT score ≤ 23 indicates clinical dementia

Figure 3 displays mean, and SEM for the mCRT total score by age group. Mean mCRT total score and age were significantly negatively associated (ρ = –0.9048 and *P* = 0.0123). The highest achieved mean mCRT score was 34.35 and corresponded to individuals aged 30 to 34. Between the ages of 40 and 44, mean mCRT was 29.67 (SD = 8.95, SEM = 0.150). Participants aged 55 to 59 had the lowest mean score, which was 17.77 (SD = 12.10, SEM = 0.275).

Table 2 shows mean mCRT and clinical diagnosis by amyloid positivity. Mean mCRT decreases with amyloid positivity. Amyloid‐negative participants had mCRT of 34.25, 34.00 at 25 Cl, 27.0 at 25 Cl, and 21.5 at 30 Cl. In addition, prevalence of CS who are amyloid negative is 95.50%, 100% at 20 CL, 100% at 25 Cl, and 54.93% at 30 Cl.

**TABLE 2:** Mean mCRT and clinical diagnosis by amyloid positivity.

	A‐	A+ (20 Cl)	A+ (25 Cl)	A+ (30 Cl)
*n*	142	4	7	72
Mean mCRT	34.25	34.00	27.00	21.50
Std mCRT	15.16	1.41	12.22	13.00
CS	95.50	100	100	54.93
MCI	1.81	0	0	19.71
DEM	0	0	0	16.90
Unable to determine	2.72	0	0	8.45

Abbreviations: Cl, Centiloids; CS, cognitively stable; DEM, dementia due to Alzheimer's disease; MCI, mild cognitive impairment; mCRT, modified Cued Recall Test.

### 3.4 Prevalence of clinical status by age

**FIGURE 4:**
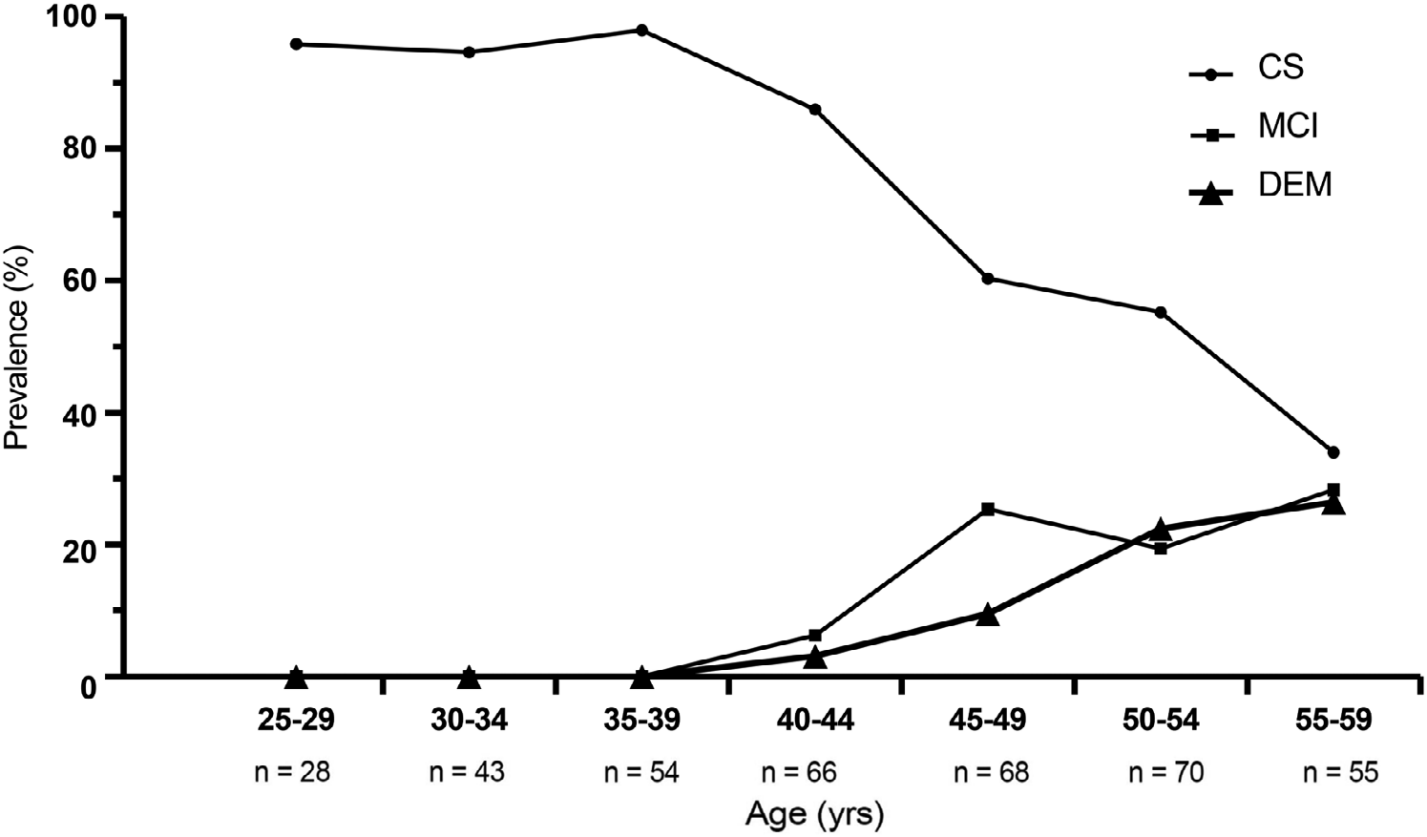
Prevalence in sampled population of clinical diagnosis (cognitively stable [CS], mild cognitive impairment [MCI], and dementia [DEM])

Figure 4 displays prevalence of clinical status by age group. Only 55.2% of participants were CS after age 50 years (i.e., in the 50‐ to 54‐year‐old and 55‐ to 60‐year‐old groups). Notably, there is only a slight decrease (~10%) in prevalence of CS status for individuals aged 25‐29 compared to 40‐44 years of age. For age cohorts ≥ 45 years old, there is a greater decline in CS individuals with an increase in percentage of participants with an MCI or DEM diagnosis. At 40 to 44 years, 3.13% of participants met clinical criteria for DEM diagnosis, while 26.4% of participants met DEM criteri between 55 and 59 years old.

## 4 DISCUSSION

The goal of this paper was to provide age‐related natural history data on clinical and imaging measures associated with the progression of AD that are likely to guide inclusion criteria for clinical trials targeting amyloid in individuals with DS. In line with prior research, age was strongly associated with both clinical and imaging biomarkers of AD; A+ prevalence and mean Aβ load increased with age, whereas mean mCRT score and prevalence of cognitively stable individuals decreased. For studies including participants aged ≥ 60, we recommend controlling for confounding effects caused by brain atrophy and AD disease progression as AD‐associated brain atrophy may affect data, namely PET Aβ load. In addition, MRI‐independent methods for amyloid PET analysis could be useful in such cases.

Differences in A+ prevalence at tested thresholds, 20, 25, and 30 Cl, demonstrate the importance of amyloid PET threshold consideration in clinical trial design. As expected, lower thresholds will identify a higher prevalence of individuals with DS who are A+. A surge in Aβ positivity prevalence occurred after the age of 39 years and indicates a potentially critical time point in Aβ pathology. Thus, clinical trials targeting Aβ accumulation could consider including participants ≤ 39 years old but will need to assess for amyloid dysmetabolism using biofluid markers. Moreover, our findings indicate that ≥ 60% of individuals with DS over the age of 45 years are A+ across all thresholds. Using a threshold of 25 Cl yields ≈ 50% A+ in individuals aged 40 to 44 years. For clinical trials requiring A+ (based on 25 Cl), this means that ≈ 50% of 40‐ to 44‐year‐olds with DS would be deemed eligible upon imaging screening. Thus, understanding A+ rates by age group can focus trials in and reduce the costs of imaging large numbers of individuals with DS who would not be A+ on PET.

Additionally, the results of the current study reflect a lag between the age at which there is evidence of amyloid pathology on PET and when clinical AD symptoms (e.g., memory decline and MCI and dementia) emerge. Indeed, early intervention during the preclinical AD stage, when A+ is present but before AD symptoms, may provide the greatest chance for disease modification and efficacy. For individuals with DS, this may be between the ages of 35 and 50 years.

Mean PET Aβ was positively associated with age; most individuals aged ≥ 40 years surpassed the 25 Cl threshold. For participants 50 to 54 years old, mean amyloid aggregation was 52.58 Cl, more than twice the A+ threshold of 25 Cl. These data are consistent with previous findings, which support a positive correlation between Aβ aggregation and age.^12^, ^19^, ^25^, ^26^ Studies including participants aged ≥ 60 must consider the effect of brain atrophy on PET Aβ. Progression of AD is characterized not only by accumulation of Aβ plaques but also by tau pathology and brain atrophy in multiple cortical regions and increased ventricle volume.^27^, ^28^ Aβ increases with age and is sensitive to mAbs intervention; thus, using PET Aβ continues to be a valid and reliable means of measuring intervention efficacy and outcome in clinical trials.

In line with prior research^16^ the mCRT had relatively small within age group variance in the current study suggesting that it offers a reproducible measure of assessing early AD‐related memory decline in the DS population. The maximum achievable score on the mCRT is 36. In our study, the highest mean score was 34.35, while the lowest was 17.77, corresponding to age groups 30 to 34 years and 55 to 59 years, respectively. Notably, the absence of a ceiling effect reinforces the mCRT as a valid and sensitive test, measuring memory deficits in DS‐AD. Linear modeling estimates mCRT score to decrease by 5.46 points with each decade of life after the age of 25. Previous studies recommend a cutoff of 28 in screening for MCI in individuals with DS who have mild intellectual disability (IQ score between 50 and 69). In contrast, a score of ≤ 23 is the recommended threshold for diagnosis of dementia. Based on our findings, we concur with these thresholds.^17^

In clinical trials targeting Aβ in persons with DS, the mCRT could provide a useful outcome measure, determining the degree to which drug therapy affects cognition in AD. Furthermore, the use of the mCRT in these trials could potentially provide evidence linking Aβ load and cognitive decline due to AD.

Analysis of clinical diagnosis status indicates that 85% of individuals with DS continue to be cognitively stable up to 44 years. Indeed, between the ages of 25 and 44 years, only 10% of individuals with DS had MCI or DEM. However, after this age point, there appears to be a steep increase in the transition to MCI and DEM. The percentage of CS individuals with DS dropped to 60.31% by age 45 years, 55.22% by age 50 years, and 33.91% by age 55 years. This trend highlights the strong correlation between age and the prevalence of clinical symptoms in AD in DS. This characterization of the prevalence of MCI and DEM by age can help guide clinical trials in selecting participant age criteria and in evaluating efficacy.

Several limitations of this study should be acknowledged. First, there were fewer participants in the older age groups, relative to the younger age groups. This difference reflects that with advanced age, more adults with DS are deceased and/or have dementia, limiting research participation. However, it is possible that confounding factors also influenced our age group sample size differences. Second, this is a cross‐sectional study, representing one point in a participant's AD progression. Future longitudinal studies are needed to define individual changes in the progression of AD and to assess how other comorbidities affect this progression. The mCRT was selected for this analysis as it has been recently shown to have excellent accuracy in capturing AD‐related cognitive decline in adults with DS.^16^, ^29^ Moreover, it is available in both English and Spanish and may represent a test that could be used in clinical practice. In this study, we focused on amyloid PET. Future studies will need to examine other ATN biomarkers (e.g., tau PET pathology) as they also have relevance for future clinical trials (e.g., anti‐tau therapeutics).

Overall, we suggest that screening and enrollment criteria in future clinical trials designed specifically for individuals with DS consider the progression of AD through biomarkers and cognitive ability across ages. Therapeutic interventions before the age of 40 years could prove to be an integral step in trials using mAbs but will need to consider very low A+ prevalence on PET. On the other hand, A+ PET prevalence increases dramatically, and well before the mean age of AD clinical diagnosis (54 years), reinforcing the notion that the preclinical AD stage (i.e., evidence of elevated brain amyloid but with a cognitive status of CS), is a prime target for secondary prevention trials of AD in people with DS.

## CONFLICT OF INTEREST STATEMENT

Author disclosures are available in the supporting information.

## CONSENT STATEMENT

Institutional review board approval and informed consent were obtained during enrollment into the study by the participant or legally designated caregiver according to the Declaration of Helsinki.

## APPENDIX‐COLLABORATORS

Alzheimer's Biomarker Consortium–Down Syndrome (ABC‐DS) Investigators:

Howard J. Aizenstein, MD, PhD; Beau M. Ances, MD, PhD; Howard F. Andrews, PhD; Karen Bell, MD; Rasmus M. Birn, PhD; Adam M. Brickman, PhD; Peter Bulova, MD; Amrita Cheema, PhD; Kewei Chen, PhD; Bradley T. Christian, PhD; Isabel Clare, PhD; Lorraine Clark, PhD; Ann D. Cohen, PhD; John N. Constantino, MD; Eric W. Doran, MS; Anne Fagan, PhD; Eleanor Feingold, PhD; Tatiana M. Foroud, PhD; Benjamin L. Handen, PhD; Jordan Harp, PhD; Sigan L. Hartley, PhD; Elizabeth Head, PhD; Rachel Henson, MS; Christy Hom, PhD; Lawrence Honig, MD; Milos D. Ikonomovic, MD; Sterling C Johnson, PhD; Courtney Jordan, RN; M. Ilyas Kamboh, PhD; David Keator, PhD; William E. Klunk, MD PhD; Julia K. Kofler, MD; William Charles Kreisl, MD; Sharon J. Krinsky‐McHale, PhD; Florence Lai, MD; Patrick Lao, PhD; Charles Laymon, PhD; Joseph Hyungwoo Lee, PhD; Ira T. Lott, MD; Victoria Lupson, PhD; Mark Mapstone, PhD; Chester A. Mathis, PhD; Davneet Singh Minhas, PhD; Neelesh Nadkarni, MD; Sid O'Bryant, PhD; Melissa Parisi, MD, PhD; Deborah Pang, MPH; Melissa Petersen, PhD; Julie C. Price, PhD; Margaret Pulsifer, PhD; Michael S. Rafii, MD, PhD; Eric Reiman, MD; Batool Rizvi, MS; Herminia Diana Rosas, MD; Laurie Ryan, PhD; Frederick Schmitt, PhD; Nicole Schupf, PhD; Wayne P. Silverman, PhD; Dana L. Tudorascu, PhD; Rameshwari Tumuluru, MD; Benjamin Tycko, MD, PhD; Badri Varadarajan, PhD; Desiree A. White, PhD; Michael A. Yassa, PhD; Shahid Zaman, MD, PhD; Fan Zhang, PhD.

## Supporting information

Supporting Information
